# Design strategies for the development of a Pd-based acetylene hydrochlorination catalyst: improvement of catalyst stability by nitrogen-containing ligands†

**DOI:** 10.1039/c9ra02572c

**Published:** 2019-07-11

**Authors:** Haihua He, Jia Zhao, Bolin Wang, Yuxue Yue, Gangfeng Sheng, Qingtao Wang, Lu Yu, Zhong-Ting Hu, Xiaonian Li

**Affiliations:** Industrial Catalysis Institute, Laboratory Breeding Base of Green Chemistry-Synthesis Technology, Zhejiang University of Technology Hangzhou 310014 China jiazhao@zjut.edu.cn xnli@zjut.edu.cn +86 571 88320002; Pharmaceutical and Material Engineering School, Jin Hua Polytechnic Jinhua 321007 China; College of Environment, Zhejiang University of Technology Hangzhou 310014 China

## Abstract

Acetylene hydrochlorination is an attractive chemical reaction for the manufacture of polyvinyl chloride (PVC), and the development efforts are focused on the search for non-mercury catalyst systems. Supported Pd-based catalysts have relatively high activity in the catalytic hydrochlorination of acetylene but are still deactivated rather quickly. Herein, we demonstrated that the atomically dispersed (NH_4_)_2_PdCl_4_ complex, distributed on activated carbon, enabled the highly active and stable production of the vinyl chloride monomer (VCM) through acetylene hydrochlorination under low temperature conditions. We found that the presence of nitrogen-containing ligands in the structure of the active center could remarkably improve the stability of the Pd-based catalysts when compared with the case of the conventional PdCl_2_ catalyst. Further analyses *via* X-ray diffraction (XRD), transmission electron microscopy (TEM), X-ray photoelectron spectroscopy (XPS) and temperature-programmed reduction (TPR) show that the variations in the Pd dispersion, chemical state and reduction property are caused by the nitrogen-containing ligands. Temperature-programmed desorption (TPD) characterizations illustrated that the N-containing ligands over the (NH_4_)_2_PdCl_4_/AC catalyst might enhance the adsorption of HCl. These findings suggest that in addition to strategies that target the doping modification of support materials, optimization of the structure of the active center complexes provides a new path for the design of highly active and stable Pd-based catalysts.

## Abstract 2

The activation of substrates over Pd active sites and the corresponding dispersion could be enhanced by the introduction of N-containing ligands.

## Graphical abstract

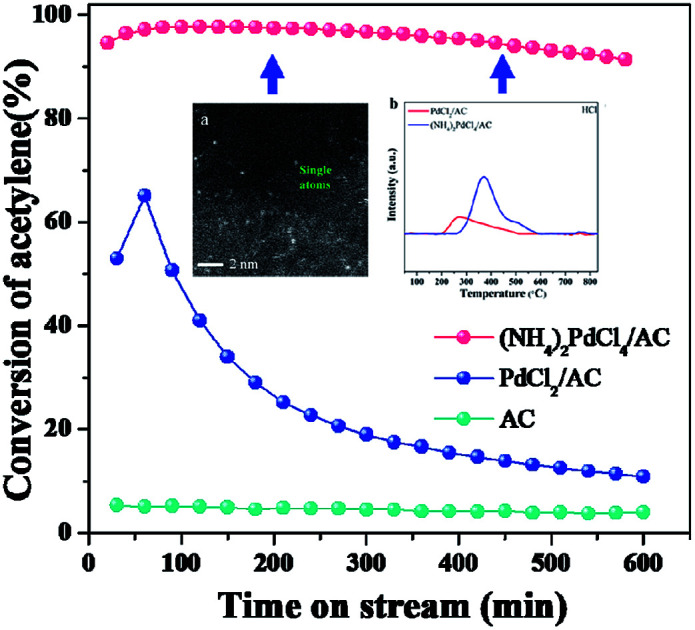

## 1 Introduction

Polyvinyl chloride (PVC) is commonly used in various manufacturing processes due to its advanced performance in mechanical enhancement, chemical inertness and stability. PVC is produced by the polymerization of the vinyl chloride monomer (VCM), synthesized either *via* the oxychlorination reaction of ethylene or *via* hydrochlorination of acetylene, and the latter approach usually employs supported mercuric chloride as a catalyst.^1–3^ However, mercuric chloride vapor may release into the environment easily under the reaction conditions and poison human beings and the environment.^4,5^ The Minamata Convention on Mercury was approved by delegates from over 140 countries in 2013 for limiting the emission and use of hazardous mercury, and now, the treaty has entered into force. Considering the current severe mercury limitations, it is urgent to develop a new mercury-free catalyst for this process.

As the selection of non-mercury catalysts poses a significant challenge, a number of catalytic formulations have been proposed including noble metal chlorides,^6–29^ non-noble metal chlorides^30–35^ and even non-metallic materials.^36–44^ Among them, Pd catalysts have been considered to be very effective for the hydrochlorination of acetylene. For example, Hutchings *et al.* explored the catalytic performance of carbon supported with a series of metal chlorides and found that the conversion of acetylene to VCM decreased in the following order: Pd(ii) > Hg(ii) > Cu(ii) ∼ Cu(i) > Ag(i) > Cd(ii) > Zn(ii).^45^ Although the Pd-based catalysts exhibited excellent hydrochlorination activity, similar to most other precious metal catalysts, they could not maintain a high level of stability. It appears that the inherent volatilization loss of the Pd active species restrains the application of Pd-based catalysts for the hydrochlorination of acetylene. Therefore, the stabilization of volatile Pd active species under the reaction conditions has become an urgent technical issue to make acetylene hydrochlorination applicable.

To date, the main strategies used for the stabilization of Pd species are still focused on the doping modification of support materials to improve the stability of the metal catalysts by enhancing the interactions of the Pd species with the supports. For example, Wang *et al.* showed that upon adjusting the surface acidity of the support, an NH_4_F-modified Pd/HY catalyst delivered enhanced stability;^46^ Bao *et al.* showed that upon doping of the carbon structure with certain nitrogen species, the stability of the PdCl_2_/AC-N catalyst was significantly improved.^47^ These experimental results suggest that one of the effective solutions to overcome the volatilization issue of Pd species is to develop a suitable support that can exhibit enhanced interaction with the Pd active centers. However, only few studies have been reported on the optimization of the structure of the active center to enhance the catalytic performance of Pd-based catalysts. Recently, it has been found that the coordination of an ionic liquid to the Pd active center can stabilize the Pd species against volatilization and then improve the catalytic stability.^48^ In this study, the well-defined (NH_4_)_2_PdCl_4_ complex was prepared, and an attempt was made to explore the catalytic performance of the carbon-supported (NH_4_)_2_PdCl_4_ complex catalysts. It was found that the (NH_4_)_2_PdCl_4_ active center could significantly stabilize the Pd species. Furthermore, the stabilization mechanism of Pd species in the (NH_4_)_2_PdCl_4_/AC catalyst was proposed on the basis of TEM, XPS, ICP, H_2_-TPR and TPD characterization. To the best of our knowledge, this is the first study that reports the use of nitrogen-containing Pd complexes in the preparation of supported Pd-based catalysts for acetylene hydrochlorination.

## 2 Experimental

### 2.1 Catalyst preparation

The (NH_4_)_2_PdCl_4_ complex was synthesized according to the literature.^49,50^ The representative procedures were conducted as follow: 35.3 mg H_2_PdCl_4_ (Sigma-Aldrich) and 23 mg NH_4_Cl (Sigma-Aldrich) were dissolved in 8 ml water at 80 °C *via* ultrasonication for 60 min to obtain yellow-brown (NH_4_)_2_PdCl_4_ crystals, and then, the catalyst preparation was conducted using an incipient wetness impregnation technique; after this, 3 g of active carbon was added to the abovementioned mixture under agitation. After drying the mixture for 12 h at 110 °C in an oven under vacuum, the synthesized catalyst was obtained, named (NH_4_)_2_PdCl_4_/AC. A carbon-supported PdCl_2_ catalyst (PdCl_2_/AC) was synthesized as a reference catalyst following the abovementioned methods except for the addition of the reagent NH_4_Cl. Unless otherwise specified, the loading amount of Pd in all Pd-based catalysts was 0.5 wt%.

### 2.2 Catalyst characterization

XPS spectra were obtained by the Kratos AXIS Ultra DLD apparatus, with a monochromatized aluminum X-ray source, and the passing energy was 40 eV. The C 1s line (284.8 eV) was adopted as the corrected benchmark for all the measured spectra. The specific surface areas were measured using N_2_ adsorption–desorption at 77 K *via* the Micromeritics ASAP 2000 instrument. TEM was used to characterize the catalyst morphology and microstructures *via* the Tecnai G2 F30 S-Twin electron microscope. The TPD was conducted in a tubular quartz reactor. Herein, 75 mg of each catalyst sample was initially treated with pure C_2_H_2_ or HCl at 180 °C for 30 min; after adsorption and sweeping with pure Ar for 60 min at the gas flow rate of 30 ml min^−1^, a temperature-programmed route was carried out from 25 °C to 550 °C at the heating rate of 10 °C min^−1^.

### 2.3 Catalytic test

Activity tests for the hydrochlorination of acetylene were conducted in a heterogeneous fixed-bed reactor. Prior to the reaction, nitrogen (N_2_) was passed through the reactor for 30 min to remove any water and air remaining. Then, C_2_H_2_ (5 ml min^−1^, 1 bar) and HCl (6 ml min^−1^, 1 bar) were added to the clean and dry reactor using a mass flow controller. The output gas products were passed through a vessel with the NaOH solution to remove excess HCl. The composition of the output was analyzed by gas chromatography (GC-9790A).

## 3 Results and discussion

### 3.1 Catalytic performance of Pd-based catalysts

**Fig. 1:**
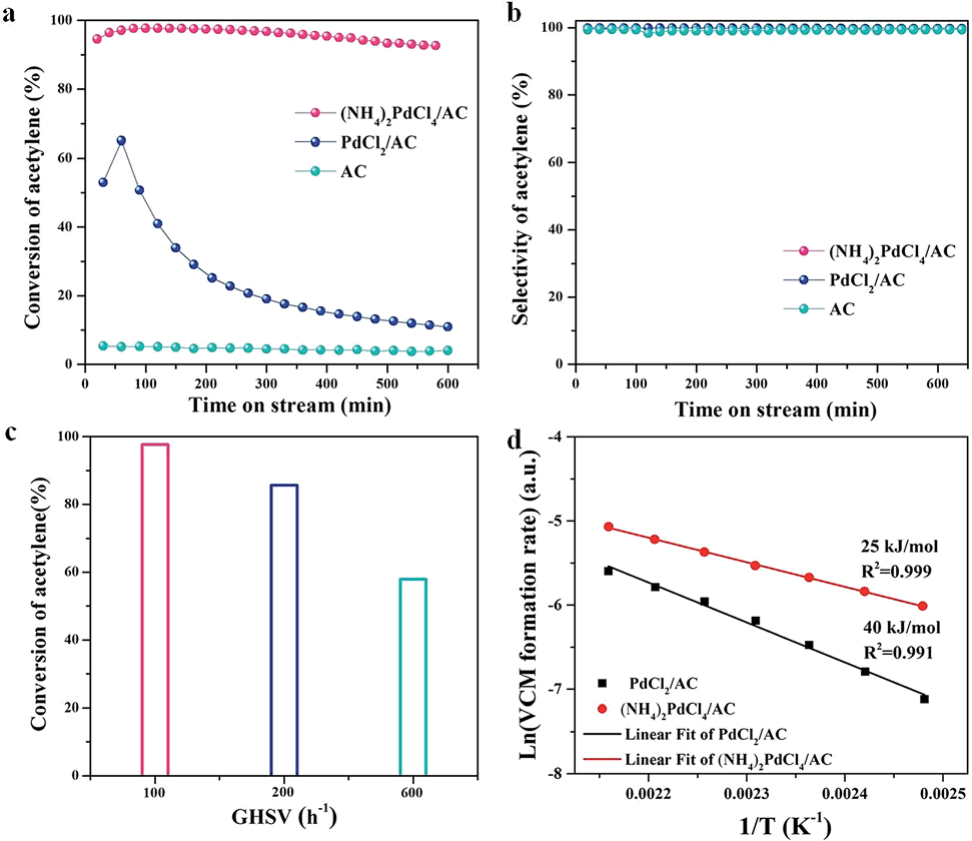
(a) Conversion of C_2_H_2_ and (b) selectivity to VCM over (NH_4_)_2_PdCl_4_/AC, PdCl_2_/AC and AC catalysts. Reaction conditions: *T* = 100 °C, GHSV(C_2_H_2_) = 100 h^−1^, and HCl/C_2_H_2_ = 1.2. (c) Catalytic activity of (NH_4_)_2_PdCl_4_/AC as a function of space velocities. Reaction conditions: *T* = 100 °C and HCl/C_2_H_2_ = 1.2. (d) Arrhenius plot for VCM formation using the (NH_4_)_2_PdCl_4_/AC and PdCl_2_/AC catalysts.

The catalytic performances of (NH_4_)_2_PdCl_4_/AC and PdCl_2_/AC catalysts are shown in Fig. 1 at the reaction temperature of 100 °C and C_2_H_2_ GHSV of 100 h^−1^. We could observe that the (NH_4_)_2_PdCl_4_/AC catalyst showed superior catalytic performance than that of the PdCl_2_/AC catalyst, resulting in a 99.7% acetylene conversion (Fig. 1a) and 99.5% VCM selectivity (Fig. 1b). Compared with PdCl_2_/AC, the (NH_4_)_2_PdCl_4_/AC catalyst also shows a remarkable robust catalytic behavior under these conditions. Note that the acetylene activity of the catalyst (NH_4_)_2_PdCl_4_ at this low temperature (100 °C) was equivalent to that of the typical Pd-based catalyst reported at high temperatures (typically exceeding 140 °C, as listed in Table S1†).

The effect of space velocity on the hydrochlorination of acetylene was further investigated. As shown in Fig. 1c, the acetylene conversion remained at 85% and 57% as the space velocity of C_2_H_2_ was increased from 100 h^−1^ to 200 h^−1^ and then to 600 h^−1^ at 100 °C, respectively. We have demonstrated that the designed (NH_4_)_2_PdCl_4_/AC catalyst is suitable for the hydrochlorination of acetylene in a wide range of space velocities.

To further clarify the structural evolution of the active phase for PdCl_2_/AC and (NH_4_)_2_PdCl_4_/AC, the kinetic behaviors were discussed by calculating the approximate activation energy (*E*_a_) (Fig. 1d). Ea was calculated based on the Arrhenius plots (ln(*R*) *versus* 1/*T*), and all the kinetic data were obtained and calculated at the level of <15% yield of vinyl chloride, which excluded the effect of internal and external diffusion. Contrary to the *E*_a_ calculated for the PdCl_2_/AC catalyst, the *E*_a_ of (NH_4_)_2_PdCl_4_/AC was markedly decreased from 40 to 25 kJ mol^−1^. The improvement in the catalytic performance may be assigned to the modulation of the intrinsic structure of the catalysts as well as the synergetic effect of Pd and the nitrogen-containing ligands on the catalytic performance.

### 3.2 Catalytic characterization

**Fig. 2:**
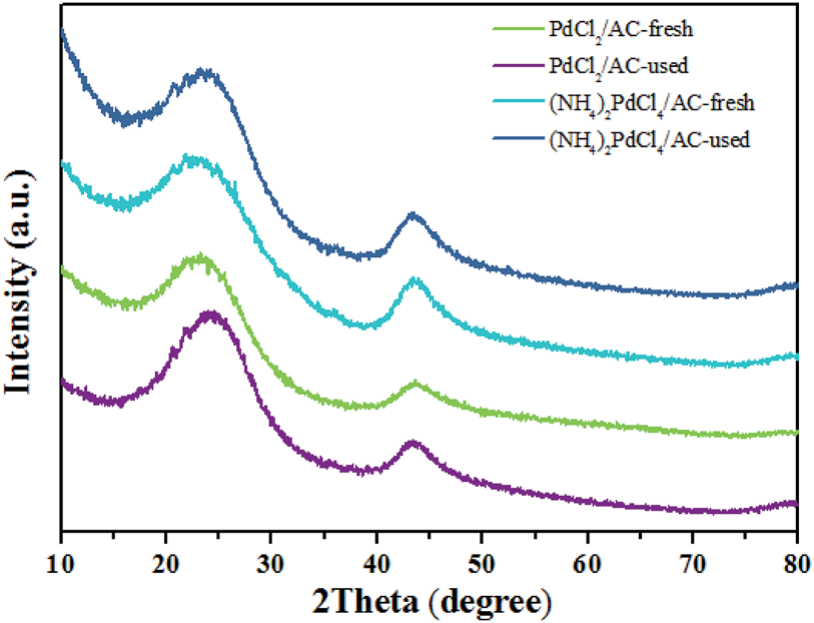
XRD patterns of the (NH_4_)_2_PdCl_4_/AC and PdCl_2_/AC catalysts.

As shown, (NH_4_)_2_PdCl_4_/AC illustrated excellent catalytic performance for the hydrochlorination of acetylene. To elucidate the influence of nitrogen-containing ligands on the catalyst microstructure, valence and adsorption properties, a series of structural characterizations were employed to analyze the Pd-based catalysts. XRD analysis was employed to probe the characteristics of the support and the Pd species. No discernible Pd^0^ characteristic diffraction peaks were observed in the XRD patterns of the fresh (NH_4_)_2_PdCl_4_/AC and PdCl_2_/AC catalysts; this suggested that most of the Pd species were present in small nanoclusters and/or non-crystalline isolated atoms (Fig. 2).

**Fig. 3:**
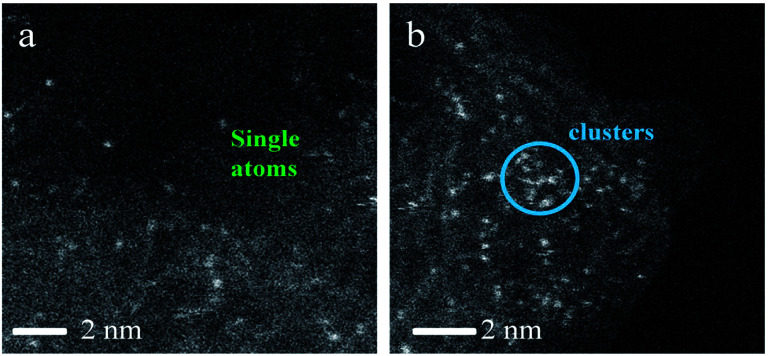
Aberration-corrected HAADF-STEM images of the (a) (NH_4_)_2_PdCl_4_/AC and (b) PdCl_2_/AC catalysts.

Fig. 3 shows an HAADF-STEM image of the (NH_4_)_2_PdCl_4_/AC and PdCl_2_/AC catalysts. As observed, the HAADF-STEM analysis clearly demonstrated the existence of isolated Pd atoms with high number density in (NH_4_)_2_PdCl_4_/AC (Fig. 3a). They are observed as bright spots evenly distributed on the surface of carbon. The light dots cannot be attributed to nitrogen species as nitrogen species cannot be distinguished from carbon in the current analysis mode. Therefore, the light dots must be assigned to Pd species. Furthermore, since the light gray dot has the typical size of 2–3 Å, these dots should be mainly assigned to single atoms/cations. For PdCl_2_/AC, it can be observed that except for the presence of isolated Pd atoms as big bright white spots, the image also illustrates the presence of Pd clusters with a few atoms (Fig. 3b). This observation provides a good reason to believe that the single isolated Pd atoms can be the active sites for the reaction, providing the observed promotion of the catalytic activity. More importantly, except for the amorphous diffraction peaks of carbon, no discernible diffraction peak was detected in the XRD pattern of the used (NH_4_)_2_PdCl_4_/AC and PdCl_2_/AC catalysts (Fig. 2); this indicated that the used Pd-based catalysts did not show the aggregation of particles.

**Fig. 4:**
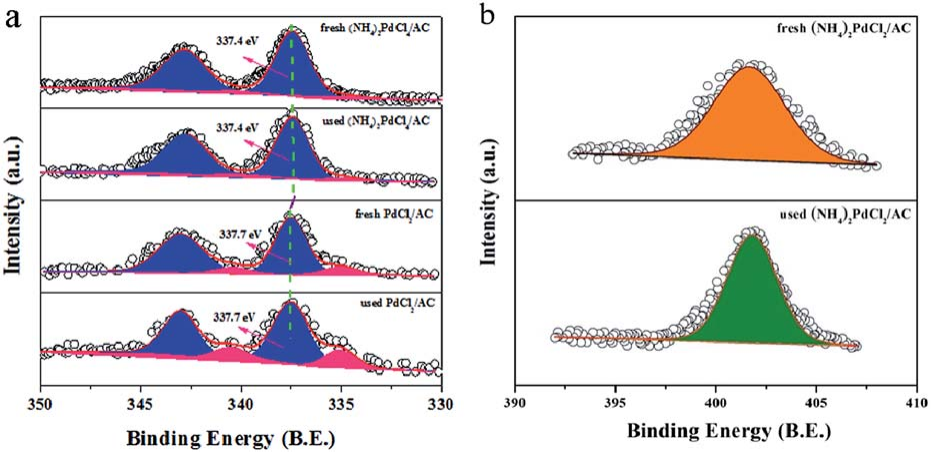
(a) XPS spectra and simulation of the fresh and used (NH_4_)_2_PdCl_4_/AC and PdCl_2_/AC catalysts. (b) N 1s spectra for the fresh and used (NH_4_)_2_PdCl_4_/AC catalyst.

Further insights into the material structure were obtained by X-ray photoelectron spectroscopy, and the deconvolution results are shown in Table 1 and Fig. 4. It is apparent that the catalyst prepared with the (NH_4_)_2_PdCl_4_ precursor presents a relatively high amount of N species (3.12%), which is absent in the PdCl_2_/AC sample; this indicates that nitrogen-containing ligands can remain stable during the preparation process. More importantly, the proportion of surface N/C has no dramatic changes before and after the reaction (Table 1 and Fig. 4b); this indicates that the (NH_4_)_2_PdCl_4_ phase does not obviously decompose during the reaction. The structure of the (NH_4_)_2_PdCl_4_ phase can remain relatively stable on the AC support. In addition, the surface content of Pd is 0.32% and 0.31% for the fresh (NH_4_)_2_PdCl_4_/AC and PdCl_2_/AC catalysts according to the XPS analysis, respectively, suggesting that the Pd species have been well dispersed on the support. After the reaction, the content of the Pd species reduces obviously to only 0.18% for PdCl_2_/AC; this may be explained by the leaching of Pd species; by contrast, there is a negligible change in the content of the Pd species for the used (NH_4_)_2_PdCl_4_/AC catalyst, which may be fairly stable in the reaction environment. Consistent with the XPS analysis, the ICP results of the fresh and used (NH_4_)_2_PdCl_4_/AC and PdCl_2_/AC catalysts also revealed that the Pd species was more stable in the presence of nitrogen-containing ligands (Table 2). Actually, the leaching of the PdCl_2_ active component was often thought to be the cause of deactivation for Pd-based catalysts in the hydrochlorination of acetylene.^44–46,51,52^ For example, Wang^41^ demonstrated that 44.7% Pd species had leached from HY zeolite-supported Pd-based catalysts after reaction; in addition, Wang suggested that the Pd loss was responsible for the deactivation of the Pd/HY catalysts, and the ICP analysis indicated that about 37.8% Pd species had been lost after 10 h time on stream as compared to the case of the fresh catalyst.

**Table 1:** Surface composition of the fresh and used (NH_4_)_2_PdCl_4_/AC and PdCl_2_/AC catalyst, determined by XPS

Catalysts	Elemental composition (wt%)			
	C	Cl	Pd	N
Fresh (NH_4_)_2_PdCl_4_/AC	94.28	2.28	0.32	3.12
Fresh PdCl_2_/AC	97.12	2.57	0.31	—
Used (NH_4_)_2_PdCl_4_/AC	93.62	3.14	0.28	2.96
Used PdCl_2_/AC	95.26	4.56	0.18	—

**Table 2:** Pd contents of the fresh and used (NH_4_)_2_PdCl_4_/AC and PdCl_2_/AC catalysts, determined by ICP

Catalysts	Nominal loading wt%	Results of ICP (wt%)		Loss ratio of Pd (%)
		Fresh	Used	
(NH_4_)_2_PdCl_4_/AC	0.5	0.52	0.46	11.5
PdCl_2_/AC	0.5	0.53	0.32	39.6

Fig. 4a displays the high-resolution spectra for the Pd 3d regions of Pd-based catalysts. The binding energies of the Pd 3d_5/2_ signals for the fresh (NH_4_)_2_PdCl_4_/AC catalyst at 337.4 eV and 335.1 eV correspond to the Pd^2+^ and Pd^0^ species (Fig. 4a).^53,54^ It is well-accepted that Pd in the cationic form works as an active species for acetylene hydrochlorination. However, the Pd 3d_5/2_ signals of the (NH_4_)_2_PdCl_4_/AC catalyst, corresponding to Pd^2+^, which represent a negative shift of 0.3 eV as compared to that of the PdCl_2_/AC catalyst (337.7 eV), demonstrate that the electronic structure of Pd changes with the incorporation of nitrogen-containing ligands. Table 3 lists the relative ratio of Pd^2+^/Pd^0^ for the fresh and used Pd-based catalysts. The results indicate that the Pd^2+^/Pd^0^ ratio in the used catalysts observably decreases when compared with that for the fresh catalysts; this indicates that the active Pd^2+^ species has been reduced under the reaction conditions. However, the (NH_4_)_2_PdCl_4_/AC catalyst experiences a slower reduction during the reaction for the Pd^2+^ species when compared with the PdCl_2_/AC catalyst. This is likely one of the reasons for the rapid deactivation observed for PdCl_2_/AC as compared to that of (NH_4_)_2_PdCl_4_/AC. Thus, nitrogen-containing ligand modalities that can enhance the stability of the Pd^2+^ species are very important for the practical applications of these catalysts.

**Table 3:** The relative ratio of Pd^2+^/Pd^0^ for Pd-based catalysts

Catalysts	Pd^2+^/Pd^0^ ratio
Fresh (NH_4_)_2_PdCl_4_/AC	14.46
Used (NH_4_)_2_PdCl_4_/AC	14.07
Fresh PdCl_2_/AC	7.54
Used PdCl_2_/AC	6.34

**Fig. 5:**
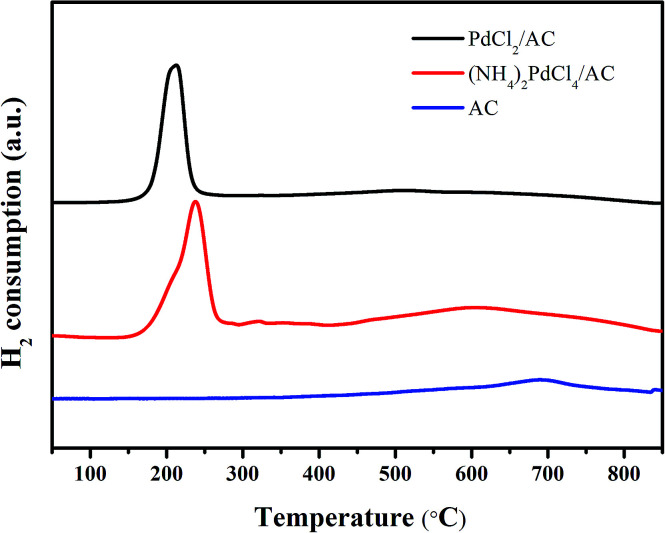
H_2_-TPR profiles of the fresh (NH_4_)_2_PdCl_4_/AC and PdCl_2_/AC catalysts.

H_2_-TPR was employed to analyse the reduction ability of the active cationic Pd species. For both PdCl_2_/AC and (NH_4_)_2_PdCl_4_/AC, a discernible characteristic reduction band in the range of 500–800 °C can be observed (Fig. 5). This band was attributed to the reduction of the surface oxygenated groups on the AC support.^55^ Apart from this, the clear bands at around 160–250 °C were due to the reduction of the cationic Pd species. The TPR profiles were analyzed by peak-differentiation-imitating analysis (Fig. S1†).^56^ Through calibration with a CuO standard, the Pd^2+^ content for the fresh catalysts can be estimated (Table S2†); the estimated Pd^2+^ contents for the fresh PdCl_2_/AC and (NH_4_)_2_PdCl_4_/AC catalysts are *ca.* 76.3% and 80.2%, respectively, which are consistent with the XPS analysis results. A more pronounced change occurred in the reduction temperature of the Pd^2+^ species. It can be seen clearly from the graph that for the (NH_4_)_2_PdCl_4_/AC catalyst, the peak for Pd^2+^ reduction is evidently increased as compared to that of PdCl_2_/AC (Fig. 5). The increased reduction temperature of (NH_4_)_2_PdCl_4_/AC can be attributed to the existence of nitrogen-containing ligands, which probably inhibit the reduction of the cationic Pd species.

The surface areas of the Pd-based catalysts were evaluated *via* the low-temperature N_2_ adsorption/desorption experiments. Table 4 lists the catalyst texture parameters of the Pd-based catalysts. It is shown that the AC support has a microporous structure, and the specific surface area is up to 1162.1 m^2^ g^−1^. Fresh catalysts, including PdCl_2_/AC and (NH_4_)_2_PdCl_4_/AC, show specific surface areas lower than that of the AC support probably due to the blockage of pores by the active Pd species. In addition, the used catalysts exhibit lower specific surface areas when compared with those of the fresh catalysts. For example, about 43.1% of the specific surface area is lost after reaction for 10 h. The loss of the active surface area may be caused by carbon deposition (acetylene may oligomerize over the catalyst) on the catalyst surface, which may result in clogged pores and decreased catalyst activity; moreover, this is likely the cause for catalyst deactivation. However, the surface area loss was only 20.8% for (NH_4_)_2_PdCl_4_/AC. This result indicates that the amount of coke deposition is significantly reduced when (NH_4_)_2_PdCl_4_ is used for Pd-based catalysts although the underlying mechanism for this reduction requires further investigation.

**Table 4:** Surface areas of the AC support and Pd-based catalysts

Catalysts	*S*_BET_ (m^2^ g^−1^)		Δ*S*_BET_ (m^2^ g^−1^)
	Fresh	Used	
AC	1162.1	—	—
(NH_4_)_2_PdCl_4_/AC	1056.1	836.5	219.6
PdCl_2_/AC	1081.3	614.4	466.9

### 3.3 Mechanism insight

**Fig. 6:**
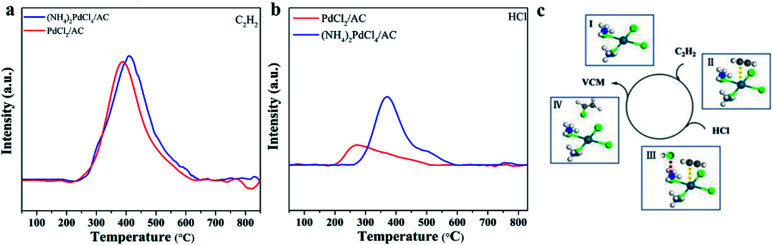
(a) C_2_H_2_-TPD and (b) HCl-TPD profiles of the fresh (NH_4_)_2_PdCl_4_/AC and PdCl_2_/AC catalysts; and (c) the proposed catalytic mechanism for the (NH_4_)_2_PdCl_4_/AC catalyst.

The adsorption and activation of substrates are important steps in a catalytic reaction. Through TPD characterization, we studied the adsorption properties of the two substrates C_2_H_2_ and HCl over the (NH_4_)_2_PdCl_4_/AC and PdCl_2_/AC catalysts. In Fig. 6, the individual C_2_H_2_-TPD results exhibit that the desorption temperature of the surveyed catalysts follows the order of PdCl_2_/AC (387 °C) < (NH_4_)_2_PdCl_4_/AC (410 °C), demonstrating that (NH_4_)_2_PdCl_4_/AC displays strong adsorption capacity for C_2_H_2_, followed by PdCl_2_/AC. The reactivity of Pd^2+^ towards C_2_H_2_ is usually explained by π-coordination and σ-coordination between the Pd^2+^ and triple bond of C_2_H_2_; thereby, C_2_H_2_ is activated. In addition, by comparing the desorption temperature of C_2_H_2_ for the two catalysts, it was found that the adsorption capacity of (NH_4_)_2_PdCl_4_/AC to C_2_H_2_ was stronger than that of PdCl_2_/AC. Similar phenomena were found in the results of HCl-TPD, where (NH_4_)_2_PdCl_4_/AC exhibited a stronger ability to absorb HCl than PdCl_2_/AC. The desorption temperatures of HCl for (NH_4_)_2_PdCl_4_/AC and PdCl_2_/AC were 369 and 264 °C, respectively. From the C_2_H_2_- and HCl-TPD results, we can observe that the desorption content of C_2_H_2_ changes negatively for (NH_4_)_2_PdCl_4_/AC and PdCl_2_/AC, whereas higher HCl desorption content has been found for (NH_4_)_2_PdCl_4_/AC than that for PdCl_2_/AC. Upon comparing the coordination structures of the Pd active sites in the catalysts (NH_4_)_2_PdCl_4_/AC and PdCl_2_/AC, it was found that the difference in the HCl desorption properties might be influenced by the presence of [NH_4_]^+^ in the (NH_4_)_2_PdCl_4_/AC catalysts. The existence of the basic ion [NH_4_]^+^ in the (NH_4_)_2_PdCl_4_ catalyst promoted the adsorption of acidic HCl molecules. In addition, because the differences between the HCl desorption temperatures and desorption contents of (NH_4_)_2_PdCl_4_/AC and PdCl_2_/AC are significantly high, HCl may be adsorbed on different sites: HCl is likely adsorbed on Pd^2+^ of the PdCl_2_/AC catalyst and [NH_4_]^+^ of the (NH_4_)_2_PdCl_4_/AC catalyst. Moreover, previous studies have shown that once the Pd-based catalyst is exposed to the feed gases, only C_2_H_2_ can be adsorbed on Pd^2+^ and HCl cannot be adsorbed due to the stronger adsorptive capacity of the catalyst for C_2_H_2_ than that for HCl. Under these conditions, the reaction follows the typical E–R mechanism for the classical PdCl_2_/AC catalyst; that is, HCl reacts with the adsorbed C_2_H_2_ on Pd^2+^ to produce vinyl chloride.

Since HCl can be adsorbed by [NH_4_]^+^, which indicates that the HCl molecule is activated during the adsorption process, there is another possibility for the mechanism of acetylene hydrochlorination over the (NH_4_)_2_PdCl_4_/AC catalyst. The possible reaction mechanism is shown in Fig. 6c. The proposed reaction mechanism was further validated by kinetic experiments. As mentioned in Fig. 1d, compared with the *E*_a_ calculated from PdCl_2_/AC, the *E*_a_ of the (NH_4_)_2_PdCl_4_/AC catalyst decreases significantly from 40 to 25 kJ mol^−1^. This is precisely due to the further activation of HCl molecules on [NH_4_]^+^ such that the energy required for the reaction becomes lower. Under the same conditions, (NH_4_)_2_PdCl_4_/AC exhibits a higher catalytic performance than PdCl_2_/AC, as shown in Fig. 1a.

## 4 Conclusions

In summary, we adopted an innovative strategy to synthesize non-mercury catalysts using the compound (NH_4_)_2_PdCl_4_ instead of traditional PdCl_2_. The prepared catalyst (NH_4_)_2_PdCl_4_/AC demonstrated excellent activity and stability in the hydrochlorination of acetylene. This indicated that the microelectronic environment of the active Pd^2+^ sites was regulated and the reduction resistance of cationic palladium was improved *via* the addition of nitrogen-containing ligands [NH_4_]^+^ to the structure of the active center. In particular, nitrogen-containing ligand additive not only can enhance the dispersion of Pd species, but can also promote the activation ability of HCl and then reduce the activation energy of the reaction. Therefore, our study proves that the (NH_4_)_2_PdCl_4_/AC catalyst can be a hopeful candidate for efficient, well-stabilized non-mercury catalysts in the manufacture of vinyl chloride.

## Conflicts of interest

There are no conflicts to declare.

## Supplementary Material

RA-009-C9RA02572C-s001
