# Supplementary material for: Design strategies for the development of a Pd-based acetylene hydrochlorination catalyst: improvement of catalyst stability by nitrogen-containing ligands
Source: RSC Adv. 2019 Jul 11;9(37):21557–63. doi: 10.1039/c9ra02572c (PMC9066347; doi:10.1039/c9ra02572c)
Supplement: RA-009-C9RA02572C-s001 [file RA-009-C9RA02572C-s001.pdf]

Electronic Supplementary Material (ESI) for RSC Advances.
This journal is © The Royal Society of Chemistry 2019

Design strategies for development of Pd-based acetylene hydrochlorination catalyst: Improvement of catalyst stability by nitrogen-containing ligands

Haihua He,[a,b] Jia Zhao,*[a] Bolin Wang,[a] Yuxue Yue,[a] Gangfeng Sheng,[a] Qingtao Wang,[a] Lu Yu,[a] Zhong-ting Hu [c] and Xiaonian Li *[a]

a. Industrial Catalysis Institute, Laboratory Breeding Base of Green Chemistry-Synthesis Technology, Zhejiang University of Technology, Hangzhou 310014, China.

b. Pharmaceutical and Material Engineering School, Jin Hua Polytechnic, Jinhua 321007, China.

c. College of Environment, Zhejiang University of Technology, Hangzhou 310014, China.

* Correspondence: jiazhao@zjut.edu.cn (Jia zhao); xnli@zjut.edu.cn (Xiaonian Li); Tel.: +86 571 88320002.

Table of contents:

Table S1. The comparisons of catalytic performance between $(NH_4)_2PdCl_4$/AC and various Pd-based catalysts reported in literatures.

Table S2. Fitting parameters of H_2-TPR profiles.

Figure S1. Procedure for the TPR area peaks determination: (a) details of the TPR profiles of the $PdCl_2$/AC catalyst (Pd^{2+} reduction) (b) the Pd^{2+} reduction peak was subject to baseline correction (c) a cumulative area counts in the interval 120 to 300 °C was carried out.

Table S1. The comparisons of catalytic performance between $(NH_4)_2PdCl_4$/AC and various Pd-based catalysts reported in literatures.

Catalysts	Pd loading (wt%)	Reaction conditions (Temperature,GHSV, V_{HCl}:V_{C2H2})	Site Time Yield (STY) ($kg_{VCM}/kg_{Metal}\cdot h$)	Reference
$PdCl_2$-KCl-$LaCl_3$/C	0.9%	180°C,120 h^{-1},1.15	7	[1]
Pd/NH_4F-HY	0.90%	160°C, 110 h^{-1}, 1.25	42	[2]
Pd-K/NFY	0.90%	160°C, 110 h^{-1}, 1.25	41	[3]
Pd/PANI-HY	0.90%	160°C, 110 h^{-1}, 1.25	40	[4]
$PdCl_2$/C	0.64%	180°C, 1080 h^{-1}, 1.1	55	[5]
Pd/HY	0.5%	160°C,110 h^{-1},1.25	61	[6]
Pd-K/HY	0.5%	160°C, 110 h^{-1}, 1.25	61	[7]
$(NH_4)_2PdCl_4$/AC	0.5%	100°C, 100 h^{-1}, 1.2	58	This work
$(NH_4)_2PdCl_4$/AC	0.5%	160°C, 370 h^{-1}, 1.2	137	This work

[a] STY ($kg_{VCM}/kg_{Metal}\cdot h$) was calculated as the average conversion of acetylene when the reaction reached steady state.

Table S2. Fitting parameters of H_2-TPR profiles.

Sample	T peak (°C)	Area (H_2)	H_2 consumed	Pd^{2+} species (%)
$PdCl_2$/AC	218	312[a]	28.3[b]	76.3
$(NH_4)_2PdCl_4$/AC	235	328	29.7	80.2

[a] The areas of the hydrogen consumption peak.

[b] The amount of H_2 consumed in μmol/g.

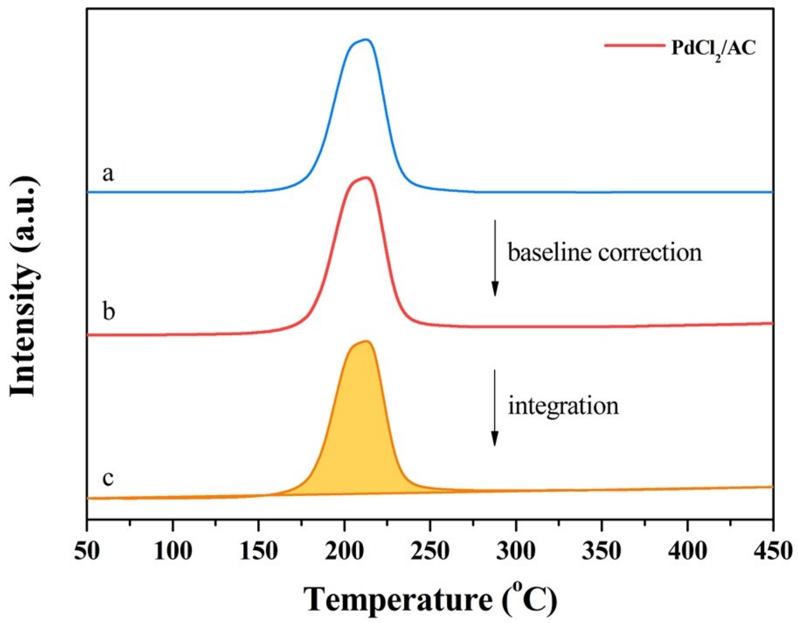

Figure S1. Procedure for the TPR area peaks determination: (a) details of the TPR profiles of the $PdCl_2$/AC catalyst (Pd^{2+} reduction) (b) the Pd^{2+} reduction peak was subject to baseline correction (c) a cumulative area counts in the interval 120 to 300 °C was carried out.

References

[1] Q. Song, S. Wang, B. Shen, J. Zhao, Petrol. Sci. Technol., 2010, 28, 1825-1833.

[2] L. Wang, F. Wang, J. Wang, Catal. Commun., 2015, 65, 41-45.

[3] L. Wang, F. Wang, J. Wang, Catal. Commun., 2016, 83, 9-13.

[4] L. Wang, F. Wang, J. Wang, Catal. Commun., 2016, 74, 55-59.

[5] B. Nkosi, N. J. Coville, G. J. Hutchings, Appl. Catal., 1988, 43, 33-39.

[6] L. Wang, F. Wang, J. Wang, X. Tang, Y. Zhao, D. Yang, F. Jia, T. Hao, React. Kine., Mech. Catal., 2013, 110 , 187-194.

[7] F. Wang, L. Wang, J. Wang, Y. Zhao, Y. Wang,; D. Yang, React. Kine., Mech. Catal., 2014, 114, 725-734.
